# The Quartet of Core Oncogenic Drivers in Neuroendocrine Prostate Cancer: Multi-Omics Dataset Integration to Forge a Translational Link Between Biology and Precision Therapy

**DOI:** 10.7150/ijbs.129521

**Published:** 2026-03-25

**Authors:** Youzhi Wang, Ning Wu, Junbo Li, Qian Cao, Jianfei Ye, Shudong Zhang

**Affiliations:** 1 Department of Urology, Peking University Third Hospital, Beijing, 100191, China.; 2 State Key Laboratory of Female Fertility Promotion, Center for Reproductive Medicine, Department of Obstetrics and Gynecology, Center for Reproductive Medicine, Peking University Third Hospital, Beijing, 100191, China.; 3 Department of Urology, Tianjin Institute of Urology, The Second Hospital of Tianjin Medical University, Tianjin, 300211, China.; 4 Department of Urology, The Affiliated Hospital of Xuzhou Medical University, Xuzhou, 221004, China.

**Keywords:** PC, NEPC, EMT, CSCs, autophagy, lineage plasticity

## Abstract

Prostate cancer (PC) is the most common cancer among American men and the second leading cause of cancer-related deaths. For advanced or metastatic PC, anti-androgen therapies, including androgen deprivation therapy (ADT), are considered standard treatment options. However, these therapies often result in the development of castration-resistant prostate cancer (CRPC) or neuroendocrine prostate cancer (NEPC), both of which present significant treatment challenge. The molecular mechanisms driving the progression from androgen - sensitive PC to castration-resistant and neuroendocrine phenotypes are still being actively investigated. This review aims to comprehensively evaluate the cellular and molecular mechanisms underlying the development of NEPC. Specifically, it will focus on the roles of cancer stem cells (CSCs), epithelial - mesenchymal transition (EMT), and autophagy in the pathogenesis and progression of NEPC. Furthermore, the review will explore the potential of targeting these processes for therapeutic intervention in advanced P. This review will integrate current findings from clinical trials, pre-clinical models, and molecular research to clarify the promising approaches for improving treatment outcomes for patients with advanced PC.

## Introduction

Prostate cancer (PC) is one of the most frequently diagnosed malignancies among men over the age of 50 in the United States and represents a growing global health burden 1-3. Despite substantial advances in therapeutic strategies, PC remains a major cause of cancer-related morbidity and mortality in urology 4-6. ADT is the cornerstone of treatment for advanced or metastatic PC. Although most patients initially respond to ADT, durable disease control is uncommon, and the majority eventually experience disease progression due to the development of therapeutic resistance 7-9. This stage of disease progression is referred to as CRPC. Resistance to androgen receptor (AR)-targeted therapy can arise through multiple mechanisms, including AR amplification or mutation, the emergence of constitutively active AR splice variants such as AR variant 7 (AR-V7), and activation of bypass signaling pathways, including glucocorticoid receptor (GR)-dependent programs 10-12. Prolonged suppression of AR signaling has also been associated with phenotypic alterations in PC cells, including lineage switching and histological dedifferentiation toward neuroendocrine differentiation (NED) and/or EMT 13-15. This adaptive process is commonly referred to as treatment-induced lineage plasticity or lineage crisis 16-18. Clinical and experimental observations indicate that prostate tumors with low AR activity often display increased cellular heterogeneity and enrichment of stem-like states, accompanied by activation of cell cycle and survival programs that contribute to aggressive clinical behavior 19, 20. Although the precise molecular mechanisms through which androgen receptor pathway inhibitors (ARPIs) promote lineage plasticity remain incompletely understood, accumulating evidence supports cellular plasticity as a key adaptive resistance strategy under sustained therapeutic pressure 21, 22.

Autophagy, a conserved cellular stress - response and recycling pathway, has emerged as an additional process that is potentially relevant to the biology of advanced PC and NEPC 23, 24. Significantly, the current evidence does not support autophagy as a unidirectional promoter or suppressor of NEPC 25, 26. Instead, it seems to function as a context-dependent, stress-adaptive process that may permissively support lineage plasticity and therapy resistance 27-29. Remarkably, autophagy has been reported to have context - dependent effects during cancer progression, acting as either a tumor-suppressive or tumor-supportive mechanism depending on the disease stage and microenvironmental conditions 30-32. In advanced tumors exposed to metabolic, oxidative, or therapeutic stress, autophagy may facilitate cellular adaptation by supporting survival, metabolic flexibility, and persistence. Nevertheless, its precise functional role in NEPC remains an area of active research 33-35.

Importantly, emerging evidence suggests that neuroendocrine prostate tumors represent a spectrum of differentiation states characterized by variable expression of neuroendocrine markers, EMT - associated features, and CSC - related properties 36, 37. Rather than functioning as independent or deterministic causal drivers, EMT programs, CSC - associated states, and autophagy - related processes are increasingly recognized as context-dependent regulatory and permissive mechanisms that may facilitate lineage plasticity, neuroendocrine differentiation, and therapy resistance during NEPC evolution 38-40.

Accordingly, this review aims to synthesize current clinical, preclinical, and molecular evidence to clarify how EMT-, CSC-, and autophagy-associated regulatory programs intersect with NEPC development and progression 41, 42. By focusing on their functional involvement within a broader network of lineage plasticity rather than proposing linear causal models, we seek to provide a balanced framework for understanding NEPC biology and for exploring potential therapeutic vulnerabilities in advanced PC 43-45.

## Mechanisms of Therapy Resistance and NE Phenotype in PC

Hormonal therapy demonstrates initial efficacy in nearly all PC patients, with chemical castration typically achieved using agents that inhibit testicular androgen secretion, such as gonadotropin-releasing hormone (GnRH) agonists or antagonists 46, 47. Nevertheless, this treatment is palliative, and castration resistance develops over time 48, 49. Despite low androgen levels, this progression primarily occurs because PC cells evade ADT by restoring AR signaling through genetic alterations 50, 51. The AR locus is associated with various AR-dependent resistance mechanisms, such as genomic amplification, AR mutations that transform antiandrogens into agonists, and AR transcription factor variants missing the carboxy-terminal ligand-binding domain, enabling AR signaling without ligand binding 52-54.

With the development of more potent ARPIs (e.g., abiraterone and enzalutamide) for CRPC treatment, additional resistance mechanisms have emerged 55, 56. For example, increased levels and/or activity of the GR enable bypass of the AR-blocking effect of enzalutamide without the need to restore AR function 57, 58. By binding to canonical AR binding sites, GR activates transcriptional programs similar to those regulated by AR 59, 60. A study reported that GR protein expression was detected in 30% (8 out of 22) of tumors following enzalutamide treatment, compared to only 10% (3 out of 22) before treatment 61, 62. Elevated GR expression following enzalutamide treatment correlated with unfavorable clinical outcomes. These data suggest that GR-mediated bypass acts as an adaptive resistance mechanism when the AR pathway is blocked 63-65.

**Figure 1:**
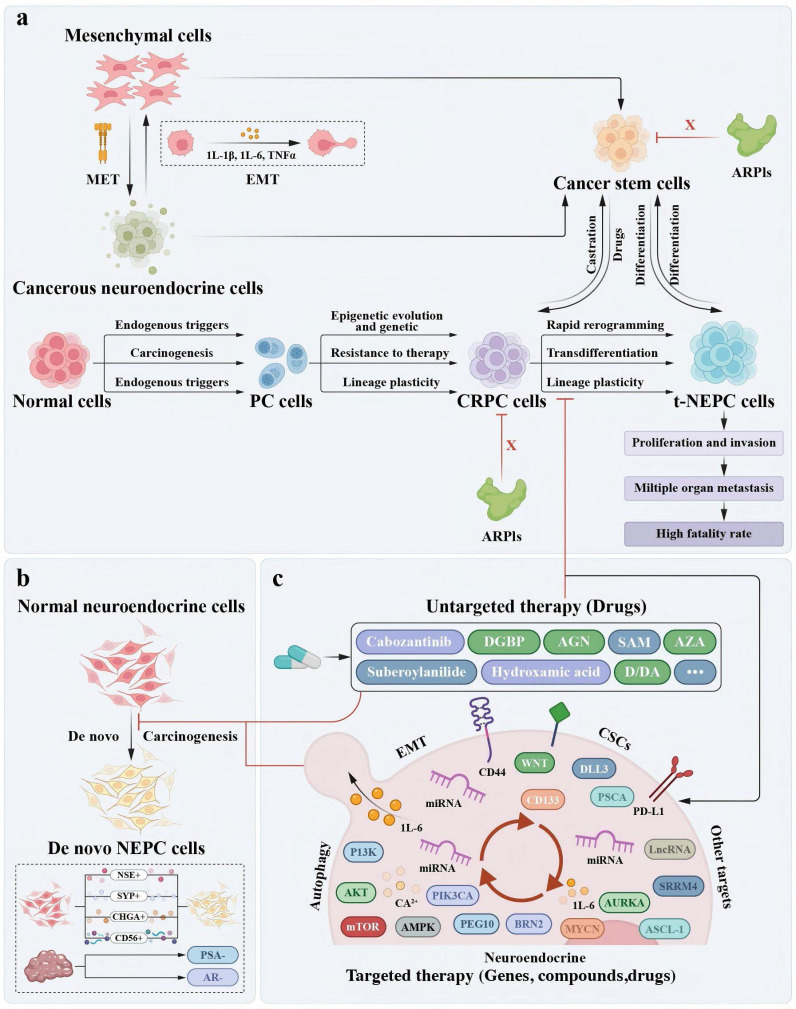
** The origin of two models of NEPC development and the diagram of integrated treatment for NE progression.** a. In the epigenetic and/or genetic dysregulation that causes PC/CRPC to transdifferentiate into the NE lineage and NEPC hierarchical model, tumours are heterogeneous, that is, they contain a mix of proliferative-type and stem-like cells. CSCs and/or NE cells cannot be eliminated by ARPIs to reduce tumour burden. These cell types can differentiate into NEPC through their plasticity. A partial EMT induces a plastic phenotype, which enables transdifferentiation to proceed via a transient pluripotent and stem-like state to generate CSCs. CRPC cells and CSCs can be interconverted by drugs and castration. Lethal NEPC induced by the interaction of cells further accelerates the proliferation and invasive activity of cancer cells and metastasis to multiple internal organs, ultimately leading to high mortality in patients. b. The *de novo* transformation of normal NE cells into NEPC. c. Mitigating the progression of NEPC by the agents that target EMT, NED, CSCs, and autophagy as well as other untargeted therapy drugs. Mitigating the progression of NEPC by other targets such lncRNA, miRNA, SRRM4, etc.

Lineage switching is recognized as a third mechanism for PC cells to evade AR pathway inhibition. In prostate cancer, 20-25% of metastatic castration-resistant prostate cancer (mCRPC) patients treated with ARPIs relapse with tumor cells exhibiting neuroendocrine characteristics, suggesting that lineage plasticity plays a role in antiandrogen resistance 66-69. Two primary hypotheses explain the origin of NEPC: one proposes that genetic or epigenetic dysregulation drives the transdifferentiation of adenocarcinomas into the NE lineage, while the other suggests that oncogenic mutations arise in normal NE cells 70-72. Regardless of its origin, NEPC's metastatic and highly aggressive nature often requires platinum-based chemotherapy, administered concurrently with small-cell carcinoma treatments 73-75. Even with chemotherapy, these tumors typically lead to patient death within two years of diagnosis (Figure 1).

## EMT, CSCs, and NED

### EMT program is associated with PC progression

Accumulating evidence indicates that PC cells frequently engage EMT-associated transcriptional and phenotypic programs that are associated with malignant traits, including enhanced invasiveness, therapeutic tolerance, and migratory capacity 76, 77. Importantly, current evidence does not support a simple linear model in which EMT-related molecules either directly induce NED or merely emerge as passive markers after NED is established 78, 79. In clinical settings, EMT-related features have been correlated with bone and lymph node metastasis, higher Gleason scores, and shortened time to biochemical recurrence, highlighting their relevance to aggressive disease phenotypes 80, 81.

Consistent with this observation, elevated expression of N-cadherin (CDH2) has been reported in CRPC cell lines and in tumors exhibiting low AR pathway activity, a feature commonly associated with AR-independent cellular states 82, 83. Functional studies further demonstrate that monoclonal antibodies targeting N-cadherin can suppress invasion, metastasis, and castration-resistant growth in CRPC xenograft models, supporting a contributory role for EMT-associated molecules in advanced disease progression 84, 85.

Therapeutic suppression of AR signaling has also been linked to changes in cellular state associated with EMT and stem-like properties 86, 87. ADT has been shown to correlate with increased expression of AR splice variants, such as AR-V7, alongside enrichment of stemness-associated markers and EMT-related transcripts 88, 89. In AR3Tg transgenic mouse models, an increased population of prostate progenitor cells within the prostate epithelium (defined as LIN⁻SCA-1⁺CD49f^high cells) has been observed, suggesting that altered AR signaling may create a permissive context for disrupted differentiation programs rather than directly inducing EMT 90, 91.

In clinical practice, circulating tumor cells (CTCs) detected in patients with CRPC undergoing treatment with enzalutamide and/or abiraterone frequently display phenotypic heterogeneity and have been associated with resistance to ARPIs 92, 93. These observations are consistent with a model in which sustained AR suppression is accompanied by the emergence or stabilization of EMT- and stemness-associated cellular states 94-96.

From a therapeutic perspective, strategies targeting AR signaling have been explored for their potential to limit these adaptive phenotypes 97, 98. Preclinical studies demonstrate that galeterone, an AR degrader, can reduce the expression of EMT- and stem cell-associated markers and exhibit antitumor activity 99, 100. However, clinical studies indicate that in patients with AR-V7-positive CRPC, galeterone did not confer a progression-free survival benefit compared with enzalutamide, underscoring the complexity of targeting EMT- and stemness-associated programs through AR-directed approaches alone 17, 101, 102.

### CSC program is associated with PC progression

Prostate stem cells (PSCs), which reside in both the luminal and basal compartments of the prostate epithelium, have been proposed as potential cells of origin for PC due to their long-lived nature and susceptibility to oncogenic alterations 103-105. In advanced disease, particularly metastatic CRPC, prostate tumors frequently harbor genetic alterations in key tumor suppressors such as TP53, RB1, and PTEN, highlighting their central role in disease progression 106-108.

Experimental studies indicate that combined genetic perturbations, rather than single events, are required to create cellular states permissive for aggressive behavior. For example, the concurrent loss of PTEN and activation of RAS signaling has been shown to induce partial EMT-like features in PSCs and CSC-enriched populations, endowing these cells with increased plasticity and metastatic potential 109-111. In contrast, PTEN inactivation alone is generally insufficient to drive metastatic progression, underscoring the importance of cooperative genetic contexts 112-114.

Importantly, the combined inactivation of PTEN, TP53, and RB1 has been consistently associated with enhanced lineage plasticity in PC cells. Rather than directly initiating NEPC, these alterations appear to establish a permissive cellular and epigenetic context in which differentiation programs are destabilized. In this setting, impaired differentiation of PSCs and CSC-enriched populations facilitates phenotypic switching, increases metastatic competence, and supports the emergence of therapy-resistant states 115, 116.

Mechanistically, TP53 and RB1 have been shown to restrain lineage plasticity, at least in part, through suppression of pluripotency-associated transcriptional programs such as SOX2. Loss of these regulatory constraints promotes stabilization of stem-like and plastic cellular states, which can subsequently cooperate with additional signaling pathways and environmental pressures to shape advanced disease phenotypes, including NEPC 117, 118.

### EMT- and CSC-associated transcriptomic programs overlap with NEPC

Multiple classical signaling pathways, including IL-6 and WNT, have been implicated in the regulation of cellular plasticity and NED in prostate cancer. Rather than operating through isolated linear cascades, these pathways converge on EMT- and CSC-associated transcriptional programs, shaping dynamic gene expression states that are permissive for lineage plasticity 119, 120. In this context, EMT- and CSC-related processes are increasingly viewed as intersecting regulatory modules that coexist with, and interact alongside, neuroendocrine transcriptional programs 121-123.

Transcriptomic analyses have revealed that EMT-associated genes, such as TWIST, SNAIL, and VIM, as well as CSC-related markers including NOTCH1, CD133, and KIT, are frequently enriched in prostate tumors exhibiting neuroendocrine features 124, 125. At the same time, canonical neuroendocrine markers—such as CHGA, SYP, and NSE, widely used in pathological and clinical settings—are elevated in NEPC. These observations support the concept that EMT, CSC, and neuroendocrine transcriptional signatures substantially overlap, rather than representing mutually exclusive or sequential states 126-128.

Several signaling mediators appear to bridge these transcriptional programs. For example, IL-6 and CD44 have been reported to correlate with EMT-associated, CSC-associated, and neuroendocrine gene expression patterns, and are consistently linked to increased cellular plasticity in PC models. Importantly, these factors are best interpreted as shared regulatory nodes within a broader plasticity network, rather than as singular determinants of neuroendocrine fate 129, 130.

To further examine the molecular intersections among EMT, CSC, and neuroendocrine transcriptional programs, we performed a systematic literature survey of PubMed-indexed studies published between 2013 and 2023 and identified 38 relevant reports (Supplementary Figure 1). Across these studies, overlapping signaling pathways, transcription factors, and cell surface receptors were repeatedly observed in models of epithelial plasticity and neuroendocrine differentiation 131-133. For instance, CD44 has been associated with enhanced NED in PC cell lines (PC3 and LNCaP) and in patient-derived NEPC tissues. Similarly, suppression of GSN was shown to reduce IL-6 expression and attenuate NED in LNCaP cells, while IL-6-associated activation of MAPK signaling facilitates formation of the TGF-β/SMAD2 complex and is linked to neuroendocrine differentiation in NCI-H660 cells 134-136.

**Figure 2:**
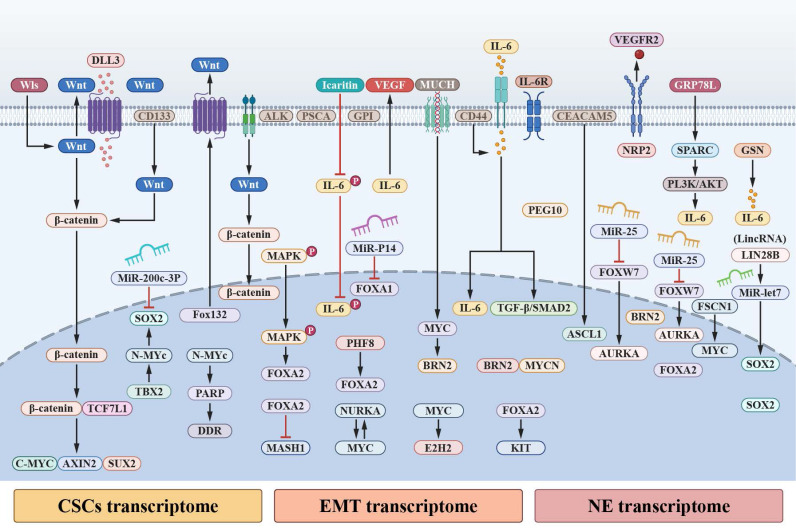
** Pivotal signalling nodes and pathways of mediating cellular plasticity and NED aggregation.** a. A convergence of WNT-β-catenin signalling pathway and IL-6 signal transducer and activator of STAT3 pathway occurs during EMT, CSCs, and NED to sustain plasticity and transcriptome dynamics. Through the interaction of IL-6 with its receptor, IL-6R, Janus Kinases (JAKs) are activated, such as JAK1 and JAK2, which in turn phosphorylate STAT3 at Tyr705; as a result, activated STAT3 dimers are translocated to the nucleus. The transcription of genes associated with NED, CSCs, and EMT are mediated by STAT3 in the nucleus by interacting with γ-activated sequences (GASs) and interferon-stimulated response elements (ISREs). Canonical WNT signalling further activate STAT3; Dishevelled (DVL) associates with the Frizzled (FZD) receptor to sequester glycogen synthase kinase-3β (GSK3β), thereby enabling the stabilization of β-catenin and its translocation to the nucleus. In the nucleus, β-catenin associates with members of the T cell factor (TCF)-lymphoid enhancer factor (LEF) transcription factor family. Activation of WNT-responsive genes is mediated by β-catenin's association with TCF-LEF transcription factors, such as STAT3. Additionally, activated FZD can activate the tyrosine-protein kinase FYN during non-canonical WNT signalling; activated FYN recruits and phosphorylates STAT3 at Tyr705 to accelerate STAT3 translocation into the nucleus and transcription. b. Collected signaling pathways converge and single or multiple regulate the three transcriptomes to influence NED and intertwine with IL-6 and WNT signaling pathways.

In cell-derived xenograft models of neuroendocrine transdifferentiation, upregulation of EMT-associated transcription factors encoded by PEG10 has been observed during the transition from adenocarcinoma to neuroendocrine-like states in LNCaP-derived and CRPC tissues 137, 138. Collectively, these findings do not support a single linear causal pathway but instead point to redundant, cooperative, and context-dependent mechanisms through which EMT-, CSC-, and neuroendocrine-associated transcriptional programs intersect to shape lineage plasticity and phenotypic diversity in advanced PC (Figure 2). In this framework, EMT-associated programs may precede, accompany, or stabilize neuroendocrine differentiation depending on genetic background, therapeutic pressure, and microenvironmental context, rather than functioning as a unidirectional trigger or a late-stage byproduct of NED 139, 140.

## Autophagy as a Stress-Adaptive Program in NEPC

### miRNA-mediated regulation of autophagy and NED

Autophagy is a conserved cellular stress-adaptation process that enables cells to maintain metabolic and proteostatic homeostasis under adverse conditions and has been reported to exert context-dependent, and sometimes opposing, effects during cancer progression 141-143. In the context of advanced PC and NEPC, autophagy has attracted increasing attention because tumor cells are frequently exposed to androgen deprivation, metabolic stress, hypoxia, and therapeutic pressure. Rather than serving solely as a housekeeping mechanism, autophagy in these settings is increasingly viewed as a context-dependent adaptive program that may support cellular persistence and phenotypic flexibility 144-146.

MicroRNAs (miRNAs) have emerged as important post-transcriptional regulators that fine-tune autophagy-related signaling networks, particularly under stress conditions relevant to lineage plasticity 147-149. Autophagy-associated genes are regulated at multiple stages—from initiation to lysosomal degradation—and numerous components of this machinery are subject to miRNA-mediated control 150, 151. Stress-induced alterations in miRNA expression can therefore modulate autophagic activity and influence downstream cellular states 152-154.

In PC models, environmental stressors such as nutrient deprivation, hypoxia, and oxidative stress—conditions frequently encountered during androgen receptor pathway inhibition—have been shown to reshape miRNA expression profiles 155, 156. These changes are closely linked to activation of autophagy-related pathways and are increasingly associated with NED and therapy-adaptive phenotypes, rather than with autophagy in isolation 157, 158.

Although thousands of miRNAs have been identified, only a subset has been implicated in autophagy regulation with relevance to prostate cancer progression 159, 160. Several miRNAs, including miR-199a-3p, miR-100, miR-128, miR-338-3p, miR-7, and miR-96, have been reported to influence autophagic activity by targeting mTOR, a central metabolic checkpoint frequently dysregulated in advanced PC 161-163. In addition, miRNA-mediated modulation of upstream regulators such as AKT signaling components, the calcium sensor CaMKKβ, and energy sensors including LKB1 and AMPK provides a mechanistic link between metabolic stress, autophagy regulation, and lineage plasticity 164, 165. Taken together, these findings suggest that miRNAs function as critical integrative nodes that connect stress-responsive autophagy programs with transcriptional and phenotypic changes associated with NEPC, rather than acting as generic regulators of autophagy alone 166, 167.

Accumulating evidence suggests that miRNAs are involved in NED-associated transcriptional reprogramming in PC, primarily by modulating stress-responsive signaling networks rather than acting as isolated initiating factors. Several miRNAs have been reported to associate with NEPC-related phenotypes and lineage plasticity in experimental and clinical contexts 168.

For example, miR-421-mediated regulation of ATM has been linked to NEPC progression, while the LIN28B/let-7/SOX2 axis has been implicated in shaping transcriptional networks associated with stem-like and plastic cellular states in NEPC 169, 170. Inhibition of miR-194 has been reported to attenuate epithelial-to-neuroendocrine-like differentiation in PC models, suggesting that specific miRNAs may modulate, rather than directly determine, neuroendocrine phenotypes. In NE-like LNCaP cells, altered expression of miR-20b, miR-106b, and miR-17 has been associated with changes in AKT3 biosynthesis, further linking miRNA dynamics to signaling adaptations observed during NED 171, 172.

Lineage switching toward NE-associated states has also been correlated with coordinated changes in miRNA expression, including upregulation of miR-301a and miR-375 and downregulation of the miR-106a~363 cluster. Importantly, these miRNA alterations are best interpreted as part of a broader transcriptional reprogramming process that accompanies NED, rather than as singular causal triggers 173.

Notably, several miRNAs implicated in autophagy regulation—such as let-7a, miR-17, miR-20a, miR-106a, miR-106b, and miR-143—have also been reported in association with NED in PC, highlighting a potential regulatory intersection between autophagy, metabolic adaptation, and lineage plasticity 174, 175. In advanced-stage TRAMP prostate tumors, AKT1 has been shown to influence the expression of multiple miRNAs (including let-7a, miR-10a, miR-143, and miR-145a), which are linked to metastatic and EMT-associated phenotypes (Table 1).

**Figure 3:**
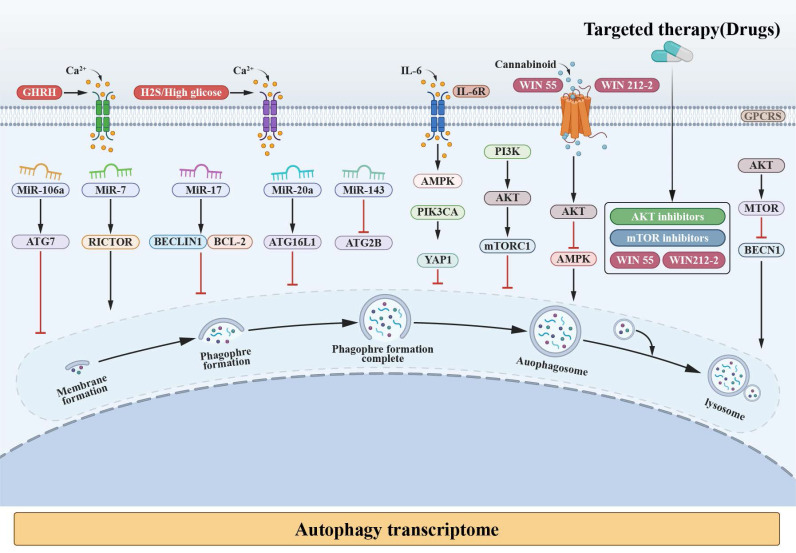
** Potential relationship between autophagy and NED in PC.** In the formation of autolysosome, an endless stream of mechanisms are involved. The key regulators, such as miRNA, PI3K-AKT-mTOR, Ca²⁺, AMPK, and PIK3CA, which rolling in the progress of autophagy and NED in PC are interviewed.

In models of neuroendocrine transdifferentiation, particularly LNCaP-derived systems, integrated analyses reveal that coordinated changes in both miRNAs and mRNAs contribute to phenotypic plasticity. Members of the miR-17 family (miR-106b, miR-106a, miR-20b, miR-17, and miR-20a) have been repeatedly highlighted in this context. Consistent with the heterogeneous nature of treatment-emergent NEPC, clinical data further indicate that CRPC-NE more frequently exhibits alterations in miR-143 compared with adenocarcinoma, while t-NEPC samples display substantial intertumoral heterogeneity, including differences in extracellular vesicle-derived miRNA profiles (Figure 3; Supplementary Figure 2).

### PI3K/AKT/mTOR-modulated autophagy under therapeutic stress

Autophagy is tightly regulated by nutrient- and energy-sensing pathways, among which the PI3K/AKT/mTOR signaling axis functions as a central metabolic checkpoint 176, 177. In advanced PC, particularly under conditions of androgen deprivation and therapeutic stress, dysregulation of this pathway has been repeatedly implicated in adaptive survival responses and lineage plasticity 178-180.

In PC models, suppression of mTORC1 activity—whether through metabolic stress, androgen receptor pathway inhibition, or pharmacologic intervention—has been consistently associated with activation of autophagy-related programs 181, 182. This response is increasingly interpreted as a stress-adaptive mechanism rather than a constitutive oncogenic driver 183, 184. In this context, autophagy may support cellular persistence by maintaining metabolic homeostasis and buffering proteotoxic and oxidative stress, conditions frequently encountered during treatment-emergent NED 185, 186.

Importantly, PI3K/AKT/mTOR signaling intersects with multiple regulatory layers relevant to NEPC biology, including EMT-associated programs, cancer stem-like states, and miRNA-mediated post-transcriptional control 187, 188. Aberrant activation of PI3K/AKT signaling—often arising from PTEN loss—has been linked to therapy resistance and lineage plasticity, while downstream modulation of mTOR activity influences autophagic flux and cellular stress tolerance 189, 190. These interactions suggest that PI3K/AKT/mTOR-regulated autophagy participates in a broader plasticity-supporting network, rather than acting as an isolated pathway 185, 186.

Although numerous signaling cascades have been reported to modulate autophagy in cancer cells, their relevance to NEPC is highly context dependent 191, 192. In this review, we focus specifically on the PI3K/AKT/mTOR axis because of its frequent alteration in advanced PC and its documented interaction with therapeutic pressure-induced phenotypic adaptation 193, 194. Pharmacological agents targeting this pathway can either enhance or suppress autophagy depending on treatment context, dosage, and cellular state, underscoring the complexity of autophagy modulation in NEPC-relevant settings (Table 2).

Collectively, current evidence supports a model in which PI3K/AKT/mTOR-regulated autophagy contributes to stress adaptation and phenotypic flexibility in advanced PC, including NEPC, rather than serving as a unidirectional driver of NED 195, 196.

Recent literature underscores the critical importance of the PI3K/AKT/mTOR signaling pathway in PC NED (Supplementary Figure 2). For instance, the lack of PKCλ/ι promotes serine production through the activation of mTORC1/ATF4, resulting in increased levels of S-adenosylmethionine, which in turn supports the development of NEPC. The cannabinoid WIN suppressed the PI3K/AKT/mTOR signaling pathway in order to modulate NEPC 197-199. Research indicates MAOA/mTOR/HIF-1α pathway that inhibiting MAOA can delay progression of NED, while hyperactive mTOR promotes NED in PC cells and is associated with increased IRF1 expression. Of the protein kinases (PKs) that showed notable alterations during NEPC transdifferentiation, 54 potentially druggable and targetable PKs were identified, primarily associated with major signaling pathways such as PI3K-AKT, MAPK, and mTOR. As previously highlighted, miRNAs and AKT are involved in a regulatory interaction during NE transdifferentiation 200-202. Research indicates that members of the miR-17 family suppress the expression of AKT3, and leading to apoptotic proteins' activation. AKT inhibitor (Triciribine), suppresses NEPC development in mice by increasing the levels of miR-669h-3p and miR-3104-3p while decreasing miR-375-3p, miR-let7a-5p, miR-10a-5p, and miR-143-3p (Figures 3) (Supplementary Figure 2).

In addition to miRNA- and PI3K/AKT/mTOR-mediated regulation, calcium (Ca²⁺)-dependent signaling has emerged as another modulatory layer linking autophagy to neuroendocrine-associated plasticity. Ca²⁺ functions as a second messenger, crucial for regulating physiological processes 203-205. Precise spatial regulation of cytosolic Ca²⁺ levels is essential for maintaining cellular homeostasis, which depends on the coordinated function of Ca²⁺ storage within organelles and the activity of multiple calcium channels, proteins, and transporters. The store-operated Ca²⁺ influx pathway, which is activated by the depletion of Ca²⁺ stores in the endoplasmic reticulum (ER), plays a crucial role in preserving Ca²⁺ balance and has been extensively studied for its regulatory effects in various types of cells, including both excitable and non-excitable ones. Ca²⁺ is also known to modulate opposing processes such as autophagy, which enhances cell survival. The lysosome functions as a signaling hub, with Ca²⁺-mediated regulation of autophagy occurring through calcineurin and TFEB activation 206-208.

NED is associated with the advancement of PC to an androgen-resistant phenotype 209, 210. Disruption of AR signaling in PC cells induces functional T-type Ca²⁺ channels, leading to significant biochemical and morphological changes (Supplementary Figure 2). In the NCI-H660 NEPC cell line, inhibiting the calcium-sensing receptor (CaSR) decreased the expression of neuroendocrine markers 211. In PC3 and 22RV1 cells transfected with SiCaSR, NE and neuronal marker expression was reduced; conversely, CaSR activation upregulated synaptophysin and NSE expression in PC3 cells 212, 213. Increased levels of exogenous agents, such as hydrogen sulfide and glucose, have been shown to enhance the expression of functional T-type Ca²⁺ channels and Cav3.2 proteins, thereby facilitating the progression of NEPC (Figure 3).

Taken together, miRNAs, PI3K/AKT/mTOR signaling, and Ca²⁺-dependent pathways converge on autophagy as a stress-adaptive regulatory program in NEPC. Importantly, most supporting evidence is derived from experimental and preclinical models, and collectively supports a context-dependent and permissive role in neuroendocrine differentiation and therapy resistance rather than a deterministic causal driver 214-216.

### Additional molecular events related to autophagy in NEPC development

In addition to classical autophagy regulators such as Ca²⁺ channels, microRNAs, and the PI3K/AKT/mTOR signaling pathway, several other molecular factors that impact NEPC are discussed below (Supplementary Figure 2). Research has shown that under conditions of androgen depletion, the AMPK-SIRT1-NO signaling axis promotes NED in LNCaP cells, a process mediated by p38MAPK-induced IL-6 secretion 217-220. Furthermore, immunohistochemical studies have identified the presence of PIK3CA mutations in patients with NEPC (Figure 3).

### Regulating the NEPC process by focusing on the “quartet”

Targeting cellular plasticity represents a promising therapeutic approach, as it plays a key role in the emergence of aggressive NE phenotypes and the development of resistance to next-generation ARPIs 115, 221, 222. Researchers are currently exploring multiple strategies to address this challenge: promoting the re-differentiation of NE carcinoma cells into AR-dependent epithelial cells to reverse NED; selectively targeting NEPC cells; and inhibiting stem cell-related and EMT pathways to prevent the initiation of NEPC (Table 3). Moreover, ongoing studies continue to identify and validate autophagy-related modulators 156, 223-225. The involvement of signaling components such as miRNAs, PI3K/AKT/mTOR pathway, Ca²⁺ signaling, and other regulators of autophagy in NEPC is being examined using a range of genetic models and pharmacological agents capable of influencing autophagic processes via these mechanisms (Table 2).

## Conclusions

Advancements in both basic and translational PC biology have enabled an unprecedentedly detailed understanding of the mechanisms and effects of antiandrogen therapy. In terms of lineage identity, the remarkable plasticity of PC cells has provided new insights into clinically relevant challenges.

In recent years, numerous studies on NEPC regulation have emerged in the field; however, the difficulty of clinical treatment and diagnosis underscores the urgency of adjusting therapeutic strategies. Currently, research on the EMT, CSC, and NED transcriptomes that regulate the NEPC process is ongoing. We continue to expand our understanding of the mechanisms by which these three regulatory modules promote NED. Moreover, novel mechanisms involved in the regulation of NEPC—such as autophagy—have gained considerable research interest. As a well-established pathway in cell death regulation, autophagy is closely linked to the progression of NEPC, offering potential avenues for innovative diagnostic and treatment strategies. Recent evidence strongly indicates that autophagy can also crosstalk with the canonical IL-6 signaling pathway to influence the development of NEPC. Observations of this process have been made not only in experimental systems but also in clinical settings.

This study demonstrates that lineage plasticity is governed not only by highly redundant and complex EMT, CSC, and NED mechanisms but also by dysregulation and crosstalk within the autophagy network. In-depth research on lineage plasticity and autophagy, along with thorough clinical trials, is crucial for enhancing our understanding of these processes and effectively regulating cell plasticity and autophagy in PC. Importantly, each of these regulatory programs is discussed in this review as a distinct yet interconnected parameter, with clearly delineated molecular determinants and context-dependent associations with NEPC rather than as linear causal drivers.

## Supplementary Material

Supplementary figures.

## Figures and Tables

**Table 1:** MiRNA known to regulate autophagy and are associated with NEPC

miRNA	Effect on autophagy	Autophagy-related target	Autophagy-related stimuli	Cell lines	Ref
MIRLET7A	Induction	RICTOR	N.D.	MGC-803 and SGC-7901	169
MIR17	Suppression	BECLIN1, BCL-2	Paclitaxel	A549, A549-T24, H596-TxR, H1734, H1734-T5, H1299, H1299-Tx	170, 171
MIR20A	Suppression	ATG16L1	Hypoxia	RAW264.7	172
MIR106A	Suppression	ATG7	N.D.	HCT116, SW620	173
MIR106B	Suppression	ATG16L1	Escherichia coli	HCT116, SW480, HeLa, U2OS	174
MIR143	Suppression	ATG7	Hydrogen peroxide	Mice cardiac progenitor cells	175

**Table 2:** Compounds and genes known to regulate autophagy and are associated with NEPC

Compounds and genetic methods	Effect	Ref
PI3K/AKT/mTOR signaling pathway		
3-methyladenine	A PtdIns3K inhibitor that effectively blocks an early stage of autophagy by inhibiting the class III PtdIns3K, but not a specifific autophagy inhibitor. 3-MA also inhibits the class I PI3K and can thus, at suboptimal concentrations in long-term experiments, promote autophagy in some systems, as well as affect cell survival through AKT and other kinases. 3-MA does not inhibit BECN1-independent autophagy.	226, 227
10-NCP	10-(40-N-diethylamino)butyl)-2-chlorophenoxazine; an AKT inhibitor that induces autophagy in neurons.	228, 229
Akti-1/2	An allosteric inhibitor of AKT1 and AKT2 that promotes autophagy in B-cell lymphoma.	230
AZD8055	A catalytic MTOR inhibitor that acts as a potent autophagy inducer.	231
Clonidine	Activates the imidazoline receptor, which decrease cAMP in cells. An MTOR-independent inducer of autophagy.	232
Eriocalyxin B	An autophagy inducer that exerts anti-tumor activity in breast cancer by inhibition of the AKT-MTOR-RPS6KB signaling pathway.	233
ESC8	A cationic estradiol derivative that induces autophagy and apoptosis simultaneously by downregulating the MTOR kinase pathway in breast cancer cells.	234, 235
Everolimus	An inhibitor of MTORC1 that induces both autophagy and apoptosis in B-cell lymphoma primary cultures.	236
Ezetimibe	A cholesterol absorption inhibitor that acts by binding to NPC1L1, which induces autophagy via MTORC1-dependent and independent pathways. Ezetimibe also activates TFEB and could potentially exert therapeutic effects on steatohepatitis and fibrosis.	237
Fasudil	An inhibitor of ROCK (Rho associated coiled-coil containing protein kinase) enhancing autophagy via phosphorylation of MAPK8/JNK1 and BCL2, and promoting BECN1-PIK3C3/VPS34 complex formation; shRNA-mediated approaches to inhibiting ROCK have similar results.	238
KU-0063794	An MTOR inhibitor that binds the catalytic site and activates autophagy.	239
MLN4924	A small molecule inhibitor of NAE (NEDD8 activating enzyme); induces autophagy by blockage of MTOR activity via both DEPTOR and the HIF1A-DDIT4/REDD1-TSC1/2 axis as a result of inactivation of cullin-RING ligases.	240
NVP-BEZ235	A dual inhibitor of PIK3CA/p110 and the MTOR catalytic site that activates autophagy.	241
Rapamycin	Inhibits MTOR by binding to RPTOR, thus inducing autophagy, but only provides partial inhibition.	242
SMER28	An MTOR-independent inducer of autophagy.	243
TMS	Trans-3,5,4-trimethoxystilbene upregulates the expression of TRPC4, resulting in MTOR inhibition.	244
Torin1	A catalytic MTOR inhibitor that induces autophagy and provides more complete inhibition than rapamycin (it inhibits all forms of MTOR).	245
Wortmannin	An inhibitor of PtdIns 3-kinase that blocks autophagy, but is not a specific inhibitor.	246
miRNA signaling pathway		
Knockdown	This method (including miRNA, RNAi, shRNA and siRNA) can be used to inhibit gene expression and provides relatively direct evidence for the role of an autophagic component. However, the efficiency of knockdown varies, as does the stability of the targeted protein. In addition, more than one gene involved in autophagy should be targeted to avoid misinterpreting indirect effects.	247
microRNA	Can be used to reduce the levels of target mRNA(s) or block translation.	248
Other signaling pathways (calcium, AMPK, PIK3CA, etc)		
Calcium	An autophagy activator that can be released from ER or lysosomal stores under stress conditions; however, calcium can also inhibit autophagy.	249, 250
Thapsigargin	An inhibitor of the sarcoplasmic/endoplasmic reticulum Ca2+ ATPase (SERCA) that inhibits autophagic sequestration through the depletion of intracellular Ca2+ stores; however, thapsigargin may also block fusion of autophagosomes with endosomes by interfering with recruitment of RAB7, resulting in autophagosome accumulation. Long-term thapsigargin treatment may induce ER stress and a secondary stimulation of autophagy.	251, 252
Resveratrol	A natural polyphenol that induces autophagy via activation of AMPK.	253
RSVAs	Synthetic small-molecule analogs of resveratrol that potently activate AMPK and induce autophagy.	254
SBI-0206965	A highly selective ULK1 kinase inhibitor *in vitro* that suppresses ULK1-mediated phosphorylation events in cells, regulating autophagy and cell survival. This compound is also an inhibitor of AMPK, competitively inhibiting ATP binding, and also inhibiting the binding of AMPK to its substrates.	255
Butein	A plant-derived natural molecule that induces autophagy through the activation of AMPK.	256
CCCP	Carbonyl cyanide m-chlorophenylhydrazone is a prototype protonophore, uncoupler of oxidative phosphorylation that stimulates autophagy via the AMPK-ULK1 pathway or alternative pathways and mitophagy, but inhibits autophagosomelysosome fusion due to the increase of intralysosomal pH.	257
Cinacalcet HCl	A calcimimetic that increases the sensitivity of CASR (calcium sensing receptor) to extracellular calcium. In some models, cinacalcet induces the formation of GFP-LC3 puncta during starvation, whereas in others it causes an increase in LC3-II accumulation in basal and CQ conditions. In a diabetic nephropathy model, the proposed pathway through cinacalcet-induced autophagy is CAMKK2/CaMKKβ-STK11/LKB1-AMPK-PPARGC1A/PGC1α to decrease oxidative stress, which results in a decrease of apoptosis (increased BCL2:BAX ratio) and increased autophagy (increase of BECN1 and LC3-I to LC3-II conversion). Cinacalcet may have a dual effect inducing autophagosome formation and inhibiting the late steps of autophagy.	258, 259
NVP-BEZ235	A dual inhibitor of PIK3CA/p110 and the MTOR catalytic site that activates autophagy.	260, 261

Supplementary: this table is not meant to be complete, as there are many compounds and genetic methods that regulate autophagy, and new ones are being discovered routinely.

**Table 3:** Treatment for directing against drivers of cellular plasticity and neuroendocrine differentiation in prostate cancer

Inhibitor	Mechanism	Clinical status	Clinical outcome	Ref
CSC inhibitors				
Disulfiram	Inhibitor of ALDH activity, which is highly expressed in prostate CSCs.	Phase Ib trial in mCRPC and NEPC ongoing.	NA	262-264
Rovalpituzumab tesirine (Rova-T)	Antibody-drug conjugate targeting the NOTCH ligand DLL3, which is expressed on CSCs and neuroendocrine cells.	Phase I trial in DLL3-expressing solid tumours, including a dedicated NEPC arm ongoing.	NA	265, 266
IL-6-STAT3 inhibitors				
Galiellalactone	Directly inhibits STAT3 transcriptional activity and induces apoptosis in ALDH^high^ prostate CSCs.	Preclinical	NA	267, 268
Lycopene	Inhibits IL-6 signalling and decreases STAT3 expression and phosphorylation.	Phase I trial in combination with docetaxel in CRPC completed.	Well tolerated with docetaxel; correlative analysis ongoing.	132, 269, 270
Siltuximab (CNTO 328)	Chimeric monoclonal antibody that binds to and neutralizes IL-6 bioactivity and inhibits STAT3 activity.	Phase I trial in combination with Docetaxel completed.	Efficacy in CRPC	12, 271, 272
MYCN and AURKA inhibitors				
Alisertib (MLN8237)	Inhibits AURKA and disrupts the AURKA- MYCN complex to inhibit MYCN-dependent Transcription.	Phase II trial in confirmed or suspected NEPC completed.	Moderate clinical response (18 of 60 patients); clinical progression-free survival of 2.3 months in NEPC arm.	273-275
GS-5829	Inhibits BET proteins.	Phase I and II trials as a single agent and in combination with enzalutamide in mCRPC ongoing.	NA	276-278
ZEN003694	Inhibits BET proteins and induces apoptosis in NEPC cell lines.	Phase I trial as a single agent and in combination with enzalutamide in mCRPC ongoing.	NA	279-281
