# Supplementary material for: The Quartet of Core Oncogenic Drivers in Neuroendocrine Prostate Cancer: Multi-Omics Dataset Integration to Forge a Translational Link Between Biology and Precision Therapy
Source: Int J Biol Sci. 2026 Mar 25;22(7):3564–79. doi: 10.7150/ijbs.129521 (PMC13086016; doi:10.7150/ijbs.129521)
Supplement: Supplementary file 1 — Supplementary figures. [file ijbsv22p3564s1.pdf]

**Supplementary Figure 1**

**Different factors that promote and/or inhibit NED in PC.**

**Influence of CSCs, EMT, and NE transcriptomes on NED in PC. Each pathway affecting NED is further systematically classified according to the type of experiment (cell lines, patients, mice models).**

**Supplementary Figure 2**

**Autophagy related pathways and targets that promote and/or inhibit NED in PC.**

**Influence of various miRNAs, PI3K-AKT-mTOR pathway, $Ca^{2+}$ channel, AMPK, and PIK3CA on NED in PC. Each pathway affecting NED is further systematically classified according to the type of experiment (cell lines, patients, mice models).**

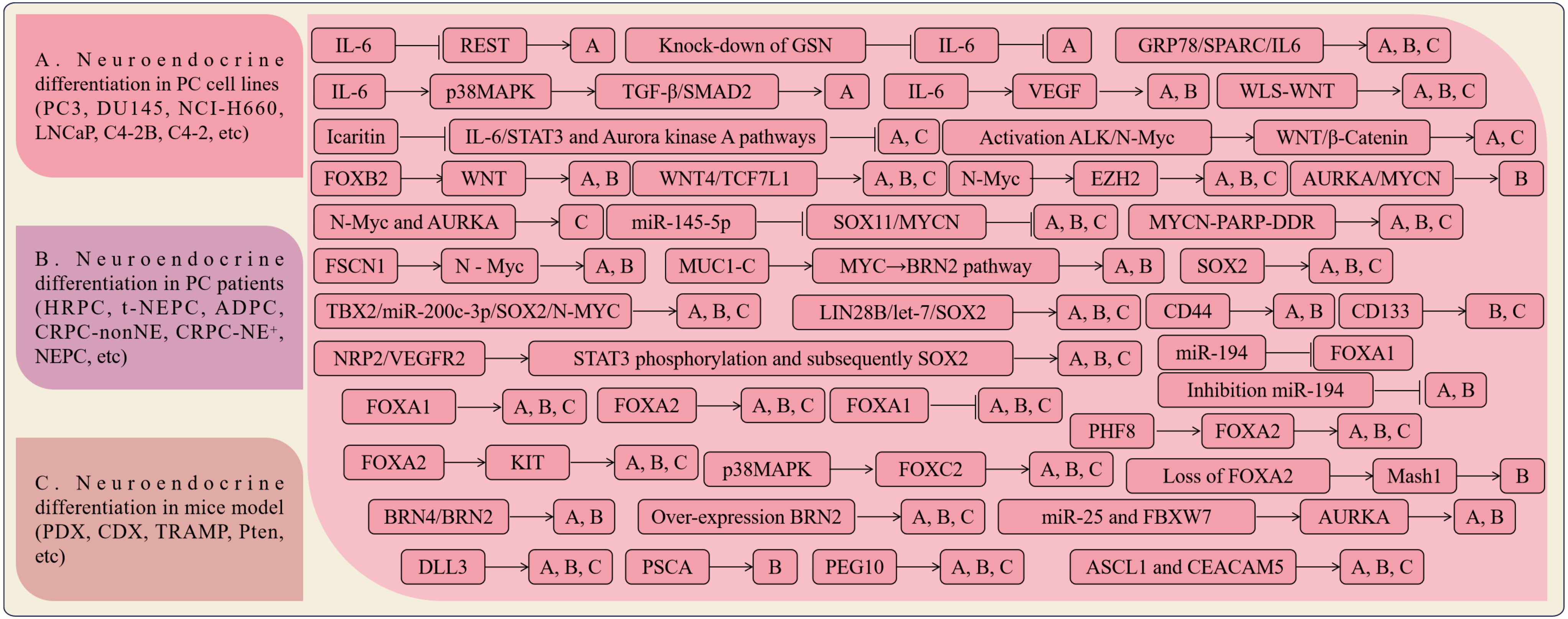
A. Neuroendocrine differentiation in PC cell lines (PC3, DU145, NCI-H660, LNCaP, C4-2B, C4-2, etc)
B. Neuroendocrine differentiation in PC patients (HRPC, t-NEPC, ADPC, CRPC-nonNE, CRPC-NE+, NEPC, etc)
C. Neuroendocrine differentiation in mice model (PDX, CDX, TRAMP, Pten, etc)
IL-6
REST
A
Knock-down of GSN
IL-6
A
GRP78/SPARC/IL6
A, B, C
IL-6
p38MAPK
TGF-β/SMAD2
A
IL-6
VEGF
A, B
WLS-WNT
A, B, C
Icaritin
IL-6/STAT3 and Aurora kinase A pathways
A, C
Activation ALK/N-Myc
WNT/β-Catenin
A, C
FOXB2
WNT
A, B
WNT4/TCF7L1
A, B, C
N-Myc
EZH2
A, B, C
AURKA/MYCN
B
N-Myc and AURKA
C
miR-145-5p
SOX11/MYCN
A, B, C
MYCN-PARP-DDR
A, B, C
FSCN1
N - Myc
A, B
MUC1-C
MYC→BRN2 pathway
A, B
SOX2
A, B, C
TBX2/miR-200c-3p/SOX2/N-MYC
A, B, C
LIN28B/let-7/SOX2
A, B, C
CD44
A, B
CD133
B, C
NRP2/VEGFR2
STAT3 phosphorylation and subsequently SOX2
A, B, C
miR-194
FOXA1
Inhibition miR-194
A, B
FOXA1
A, B, C
FOXA2
A, B, C
FOXA1
A, B, C
PHF8
FOXA2
A, B, C
FOXA2
KIT
A, B, C
p38MAPK
FOXC2
A, B, C
Loss of FOXA2
Mash1
B
BRN4/BRN2
A, B
Over-expression BRN2
A, B, C
miR-25 and FBXW7
AURKA
A, B
DLL3
A, B, C
PSCA
B
PEG10
A, B, C
ASCL1 and CEACAM5
A, B, C

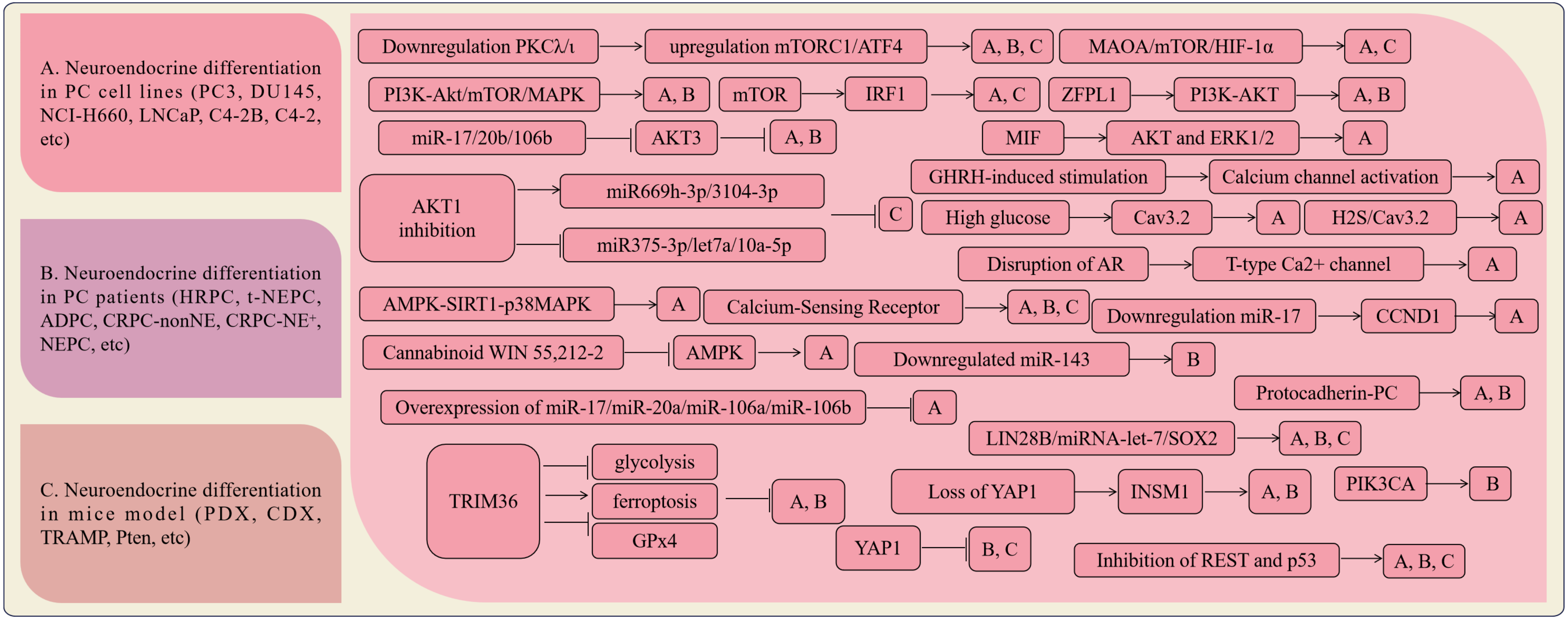

A. Neuroendocrine differentiation in PC cell lines (PC3, DU145, NCI-H660, LNCaP, C4-2B, C4-2, etc)
B. Neuroendocrine differentiation in PC patients (HRPC, t-NEPC, ADPC, CRPC-nonNE, CRPC-NE+, NEPC, etc)
C. Neuroendocrine differentiation in mice model (PDX, CDX, TRAMP, Pten, etc)
Downregulation PKCλ/ι
upregulation mTORC1/ATF4
A, B, C
MAOA/mTOR/HIF-1α
A, C
PI3K-Akt/mTOR/MAPK
A, B
mTOR
IRF1
A, C
ZFPL1
PI3K-AKT
A, B
miR-17/20b/106b
AKT3
A, B
MIF
AKT and ERK1/2
A
GHRH-induced stimulation
Calcium channel activation
A
AKT1 inhibition
miR669h-3p/3104-3p
miR375-3p/let7a/10a-5p
C
High glucose
Cav3.2
A
H2S/Cav3.2
A
Disruption of AR
T-type Ca2+ channel
A
AMPK-SIRT1-p38MAPK
A
Calcium-Sensing Receptor
A, B, C
Downregulation miR-17
CCND1
A
Cannabinoid WIN 55,212-2
AMPK
A
Downregulated miR-143
B
Protocadherin-PC
A, B
Overexpression of miR-17/miR-20a/miR-106a/miR-106b
A
LIN28B/miRNA-let-7/SOX2
A, B, C
TRIM36
glycolysis
ferroptosis
GPx4
A, B
Loss of YAP1
INSM1
A, B
PIK3CA
B
YAP1
B, C
Inhibition of REST and p53
A, B, C
